# Regulation of Brassinosteroid Homeostasis in Higher Plants

**DOI:** 10.3389/fpls.2020.583622

**Published:** 2020-09-29

**Authors:** Zhuoyun Wei, Jia Li

**Affiliations:** Ministry of Education Key Laboratory of Cell Activities and Stress Adaptations, School of Life Sciences, Lanzhou University, Lanzhou, China

**Keywords:** brassinosteroids, phytohormones, homeostasis, cytochrome P450, transcriptional regulation

## Abstract

Brassinosteroids (BRs) are known as one of the major classes of phytohormones essential for various processes during normal plant growth, development, and adaptations to biotic and abiotic stresses. Significant progress has been achieved on revealing mechanisms regulating BR biosynthesis, catabolism, and signaling in many crops and in model plant *Arabidopsis*. It is known that BRs control plant growth and development in a dosage-dependent manner. Maintenance of BR homeostasis is therefore critical for optimal functions of BRs. In this review, updated discoveries on mechanisms controlling BR homeostasis in higher plants in response to internal and external cues are discussed.

## Introduction

Brassinosteroids (BRs) are a group of naturally occurring and polyhydroxylated phytosterols, carrying at least one oxygen moiety at the C3 position and additional ones at one or more of C2, C6, C22, and C23 carbon atoms (Bishop and Yokota, 2001). Since brassinolide (BL), the most active BR compound, was first isolated from *Brassica napus* pollen grains in 1970s, more than 70 BR compounds have been identified and they are ubiquitously presented in the plant kingdom (Mitchell et al., 1970; Grove et al., 1979; Bajguz and Tretyn, 2003). It is widely known that BRs regulate multiple processes during plant growth, development and environmental adaptations, especially controlling many important agronomic traits such as plant architecture, flowering time, seed yield, and stress tolerance (Clouse and Sasse, 1998; Tong and Chu, 2018). Therefore, genetic control of endogenous BR levels or signaling offers a novel approach for crop improvement.

Although application of limited amounts of BRs can significantly enhance growth, excessive BRs are usually harmful to plant growth and development (Clouse et al., 1996). Maintenance and regulation of endogenous BR levels are therefore essential for optimal plant growth and development. Considering that BRs cannot undergo long distance transport, BR biosynthesis and catabolism are two critical antagonistic processes for maintaining BR homeostasis in plants (Symons and Reid, 2004; Ye et al., 2011; Zhao and Li, 2012). In the past decades, extensive researches have been conducted to elucidate the BR biosynthesis pathway in many plant species. Various enzymes catabolizing bioactive BRs through acylation, sulfonation, glycosylation, or other manners in these plants have also been identified. This review focuses on the recent advances in our understanding of the dynamic regulation of BR homeostasis in higher plants in response to various internal and external factors. These pieces of information can be used to facilitate BR application in molecular design for modern agriculture.

## BR Biosynthesis Pathways

BRs are classified as C27, C28, and C29 steroids based on the structure of their C24 alkyl groups (Fujioka and Yokota, 2003). C28 BRs, such as castasterone (CS) and BL, are the most abundant and ubiquitous BRs in plants. Synthesis of CS and BL from campesterol, one of the plant sterols, has been clearly elucidated and is discussed in detail. C27 and C29 BRs use two other compounds, cholesterol, and sitosterol, as their corresponding precursors, and may go through pathways similar to those of C28 BRs (Sakurai, 1999; Fujioka and Yokota, 2003).

### Sterol Biosynthesis From Cycloartenol to Campesterol and Sitosterol

#### The Common Steps

**Figure 1:**
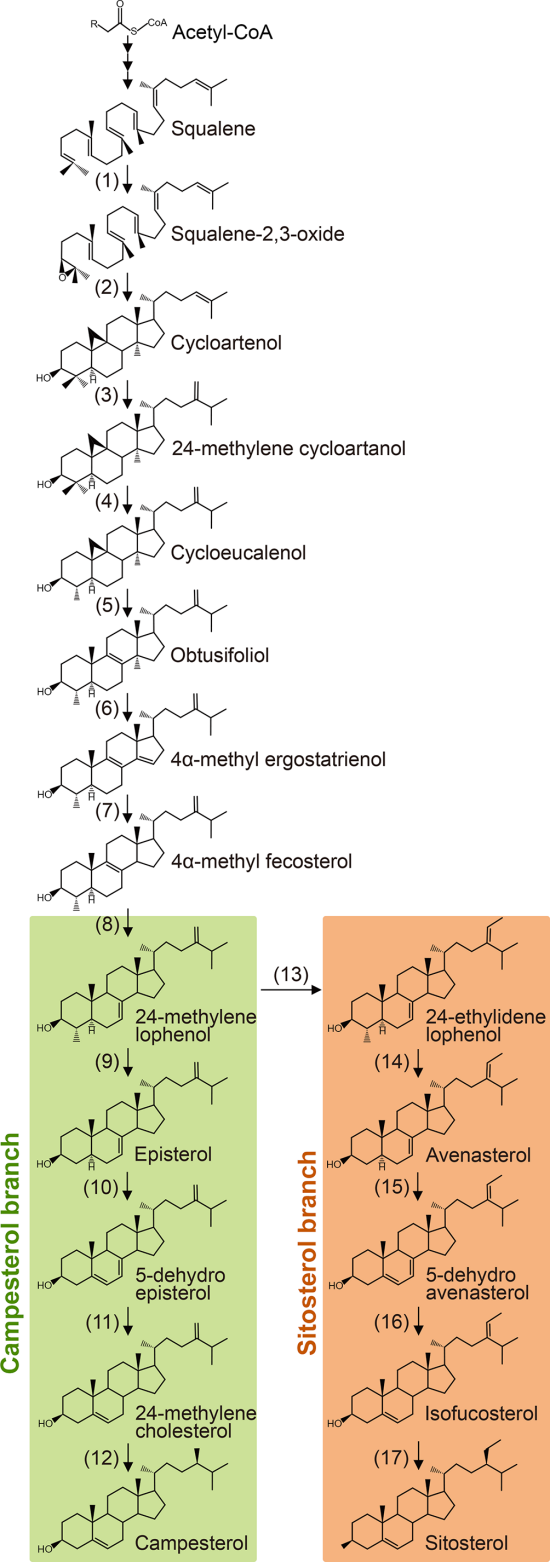
Biosynthesis of campesterol and sitosterol from squalene. Numbers correspond to the description in the text and **Table 1**. Enzymes identified from higher plants are listed in **Table 1**.

Plant sterols are synthesized from cycloartenol, a plant-specific C30 sterol derived from squalene (**Figure 1**). Most of the enzymes involved in the phytosterol biosynthetic pathway have been characterized in different plant species (**Table 1**). (1) Squalene epoxidase (SQE) catalyzes the oxidation of squalene to squalene-2,3-oxide (Rasbery et al., 2007; Pose et al., 2009; Unland et al., 2018; Liu et al., 2020a). (2) Conversion of squalene-2,3-oxide into cycloartenol is catalyzed by a cycloartenol synthase (Corey et al., 1993; Babiychuk et al., 2008; Gas-Pascual et al., 2014). (3) The first C24 methylation reaction converts cycloartenol into 24-methylene cycloartenol (Shi et al., 1996; Diener et al., 2000; Holmberg et al., 2002; Schrick et al., 2002; Willemsen et al., 2003; Guan et al., 2017). This rate-limiting methylation step leads to subsequent synthesis of 24-methyl (campesterol) or 24-ethyl (sitosterol) instead of 24-desmethyl sterol (cholesterol). The second C24 methylation reaction after several steps determines the formation of 24-ethyl sterols instead of 24-methyl sterols. (4) Cycloeucalenol is produced from 24-methylene cycloartenol through demethylation at C4 position, which is performed with the sequential participation of three enzymes, a sterol 4α-methyl oxidase (SMO), a 4α-carboxysterol-C3-dehydrogenase/C4-decarboxylase (CSD), and a sterone ketoreductase (Darnet et al., 2001; Darnet and Rahier, 2004; Rahier, 2011; Song et al., 2019). Removal of the two methyl groups at C4 position is essential for sterols to be functional. The two separate C4 demethylation reactions in higher plants are catalyzed by two distinctive families of SMO enzymes, whereas the two consecutive C-4 demethylation reactions are catalyzed by the same enzymes in animals and fungi (Rahier, 2011). (5) Cycloeucalenol is then isomerized by cyclopropyl sterol isomerase. As a result, obtusifoliol is produced (Lovato et al., 2000; Men et al., 2008). (6) Subsequently, CYP51, one of the most ancient and conserved cytochrome P450s across the kingdoms, demethylates obtusifoliol at C14 to form 4α-methyl ergostatrienol (Kahn et al., 1996; Bak et al., 1997; Cabello-Hurtado et al., 1999; Kushiro et al., 2001; Burger et al., 2003; Kim et al., 2005a). (7) C14 reduction of 4α-methyl ergostatrienol is catalyzed by FACKEL/HYDRA2/EXTRA-LONG-LIFESPAN 1 (FK/HYD2/ELL1), three alleles from *Arabidopsis* isolated by independent research groups, leading to formation of 4α-methyl fecosterol (Jang et al., 2000; Schrick et al., 2000; Souter et al., 2002). (8) Isomerization of 4α-methyl fecosterol into 24-methylene lophenol is catalyzed by a Δ^8^-Δ^7^ sterol isomerase, identified as HYDRA1 (HYD1) in *Arabidopsis* (Souter et al., 2002).

**Table 1:** Sterol biosynthesis enzymes identified in different plant species.

Function	Species	Name	Steps*	Reference
Squalene epoxidase	*Arabidopsis thaliana*	SQE1	1	Rasbery et al., 2007; Pose et al., 2009
	*Tripterygium wilfordii*	TwSQE6/7	1	Liu et al., 2020a
	*Taraxacum koksaghyz*	TkSQE1	1	Unland et al., 2018
Cycloartenol synthase	*Arabidopsis thaliana*	CAS1	2	Corey et al., 1993; Babiychuk et al., 2008
	*Nicotiana tabacum*	NtCAS1	2	Gas-Pascual et al., 2014
Sterol methyltransferase	*Arabidopsis thaliana*	AtSMT1/CPH/ORC	3	Diener et al., 2000; Schrick et al., 2002; Willemsen et al., 2003
		CVP1/SMT2/3	13	Husselstein et al., 1996; Bouvier-Navé et al., 1997; Schaller et al., 1998; Carland et al., 1999; Schaeffer et al., 2001; Carland et al., 2002; Carland et al., 2010
	*Glycine max*	SMT1	3	Shi et al., 1996
	*Nicotiana tabacum*	NtSMT1	3	Holmberg et al., 2002
	*Tripterygium wilfordii*	TwSMT1	3	Guan et al., 2017
	*Gossypium hirsuturm*	GhSMT2-1/2-2	3	Luo et al., 2008
Sterol 4α-methyl oxidase	*Arabidopsis thaliana*	SMO1	4	Darnet et al., 2001; Darnet and Rahier, 2004; Song et al., 2019
		SMO2	9/14	Zhang et al., 2016
Cyclopropyl sterol isomerase	*Arabidopsis thaliana*	CPI1	5	Lovato et al., 2000; Men et al., 2008
14α‐demethylase	*Arabidopsis thaliana*	CYP51A2	6	Kushiro et al., 2001; Kim et al., 2005a
	*Oryza sativa*	OsCYP51G3	6	Xia et al., 2015
	*Sorghum bicolor*	CYP51	6	Kahn et al., 1996; Bak et al., 1997
	*Triticum aestivum*	CYP51	6	Cabello-Hurtado et al., 1999
	*Nicotiana benthamiana*	CYP51	6	Burger et al., 2003
C14 reductase	*Arabidopsis thaliana*	FK/HYD2/ELL1	7	Jang et al., 2000; Schrick et al., 2000; Souter et al., 2002
Δ8-Δ7 sterol isomerase	*Arabidopsis thaliana*	HYD1	8	Souter et al., 2002
C5 desaturase	*Arabidopsis thaliana*	DWF7/STE1/BUL1	10/15	Gachotte et al., 1995; Choe et al., 1999b; Catterou et al., 2001a; Catterou et al., 2001b
C7 reductase	*Arabidopsis thaliana*	DWF5	11/16	Choe et al., 2000
Δ24 isomerase/reductase	*Arabidopsis thaliana*	DWF1/CBB1/DIM	12/17	Takahashi et al., 1995; Kauschmann et al., 1996; Klahre et al., 1998; Choe et al., 1999a
	*Oryza sativa*	BRD2/LTBSG1/LHDD10	12/17	Hong et al., 2005; Liu et al., 2016; Qin et al., 2018
	*Pyrus ussuriensis*	PcDWF1	12/17	Zheng et al., 2020
	*Hordeum vulgare*	HvDIM	12/17	Dockter et al., 2014
	*Zea mays*	NA2	12/17	Best et al., 2016

*Step numbers correspond to the description in the text and **Figure 1**.

#### The Campesterol Branch

(9) Removal of the second methyl group at C4 converts 24-methylene lophenol into episterol, which involves a family of SMO enzymes distinctive from the first C4 demethylation reaction (Zhang et al., 2016). (10) Episterol is subsequently converted into 5-dehydro episterol by a C5 desaturase, named DWARF7/STE1/BOULE1 (DWF7/STE1/BUL1) in *Arabidopsis* (Gachotte et al., 1995; Choe et al., 1999b; Catterou et al., 2001b; Catterou et al., 2001a). (11) C7 reductase, also designated as DWARF5 (DWF5) in *Arabidopsis*, reduces 5-dehydro episterol to yield 24-methylene cholesterol (Choe et al., 2000). (12) The Δ^24(28)^ bond of 24-methylene cholesterol is isomerized into a Δ^24(25)^ bond, and then the double bond is reduced to produce campesterol, the specific precursor of BR biosynthesis. Both the isomerization and reduction are catalyzed by a single enzyme, named as DWF1/CBB1/DIM and BRD2/LTBSG1/LHDD10 in *Arabidopsis* and rice, respectively (Takahashi et al., 1995; Kauschmann et al., 1996; Klahre et al., 1998; Choe et al., 1999a; Hong et al., 2005; Best et al., 2016; Liu et al., 2016; Qin et al., 2018; Zheng et al., 2020).

#### The Sitosterol Branch

(13) The first step of sitosterol branch is the second C24 methylation reaction in the plant sterol biosynthesis pathway that converts 24-methylene lophenol into 24-ethylidene lophenol, the fundamental member of 24-ethyl sterols (Husselstein et al., 1996; Bouvier-Navé et al., 1997; Schaller et al., 1998; Carland et al., 1999; Schaeffer et al., 2001; Carland et al., 2002; Luo et al., 2008; Carland et al., 2010). (14-17) Subsequent four consecutive steps, including C4 demethylation, C5 desaturation, C7 reduction, and C24 isomerization/reduction, leads to the final biosynthesis of sitosterol. These four steps are catalyzed by the same enzymes functioning in the parallel campesterol branch (Klahre et al., 1998; Choe et al., 1999b; Choe et al., 2000; Zhang et al., 2016).

### Specific Biosynthesis of BL From Campesterol

**Figure 2:**
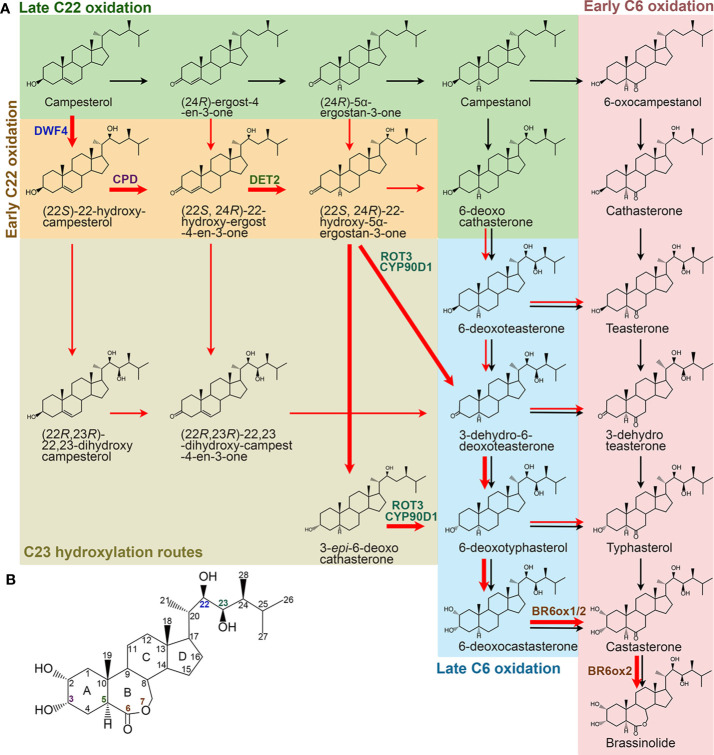
Specific BR biosynthetic pathways from campesterol in higher plants. **(A)** Black and red arrows represent CN-dependent and -independent pathways, respectively. Bold red arrows represent the most dominant and efficient flow of the BR intermediates. Enzymes identified from Arabidopsis are only shown in the dominant routes. **(B)** Molecular structure of BL. The carbons are numbered and the rings are labelled by letters. Colors of the number correspond to the enzymes shown in **(A)**.

BR biosynthesis involves parallel and highly networked pathways (**Figure 2**). Campesterol can be first converted into campestanol (CN) through a late C22 oxidation pathway. CN in turn is converted to CS either *via* an early C6 oxidation or a late C6 oxidation pathway, which is also called a CN-dependent pathway (Fujioka and Yokota, 2003). On the other hand, campesterol can be first converted into 6-deoxocathasterone through an early C22 oxidation pathway, either flowing straightly or *via* a C23 hydroxylation reaction step then going into the C6 oxidation pathways to synthesize CS, which is also designated as a CN-independent pathway (Fujioka et al., 2002; Fujita et al., 2006; Ohnishi et al., 2006b). CS is the end and most bioactive BR compound in graminaceous plants, such as rice (Kim et al., 2008). Whereas, CS can further be converted into BL in most dicotyledonous plants due to the duplication of a C6 oxidase gene, one of their encoded C6 oxidases developed a BL synthase function (Kim et al., 2005b; Nomura et al., 2005). Compared with their parallel branches, the early C22 oxidation pathway and the late C6 oxidation pathway appear to be the predominant route in many plant species, including *Arabidopsis*, tomato, and pea (Nomura et al., 2001; Fujioka et al., 2002). Furthermore, the C23 hydroxylases prefer to use (22*S*, 24*R*)-22-hydroxy-5α-ergostan-3-one and 3-*epi*-6-deoxocathasterone as their substrates (Ohnishi et al., 2006b). Thus, the most dominant and efficient flow of BR intermediates, campesterol → (22*S*)-22-hydroxy-campesterol → (22*S*, 24*R*)-22-hydroxy-ergost-4-en-3-one → (22*S*, 24*R*)-22-hydroxy-5α-ergostan-3-one → 3-*epi*-6-deoxocathasterone/3-dehydro-6-deoxoteasterone → 6-deoxotyphasterol → 6-deoxocastasterone → CS → BL, is established (Ohnishi et al., 2012). Although there are two more steps in other biosynthetic routes compared with this dominant CN-independent pathway, all the BR biosynthesis routes involve common reaction steps, including hydroxylation at C22, C23, and C2, oxidation and reduction at C3, reduction at C5, and oxidation at C6, and an additional Baeyer-Villiger oxidation in most dicotyledonous plants. Most of the enzymes involved in the reactions were identified in different plant species (**Table 2**). Loss of function of these enzymes leads to similar defective phenotypes, including dwarf and compact plant architecture, short roots, delayed flowering time, reduced biomass and seed yield.

**Table 2:** Specific BR biosynthesis enzymes identified in different plant species.

Function	Species	Name	Reference
C22 hydroxylase	*Arabidopsis thaliana*	DWF4/CYP90B1	Choe et al., 1998; Fujita et al., 2006
		CYP724A1	Zhang et al., 2012
	*Oryza sativa*	CYP90B2/OsDWF4	Sakamoto et al., 2006
		CYP724B1/D11*	Sakamoto et al., 2006
	*Lycopersicon esculentum*	CYP90B3	Ohnishi et al., 2006c
		CYP724B2	Ohnishi et al., 2006c
	*Zea mays*	CYP90B2/ZmDWF4	Liu et al., 2007
	*Solanum tuberosum*	StDWF4	Zhou et al., 2018
	*Populus tomentosa*	PtoDWF4	Shen et al., 2018
C23 hydroxylase	*Arabidopsis thaliana*	CYP90C1/ROT3 CYP90D1	Ohnishi et al., 2006b
	*Oryza sativa*	CYP90D2/OsD2* CYP90D3	Sakamoto et al., 2012
	*Lycopersicon esculentum*	DPY	Koka et al., 2000
C2 hydroxylase	*Pisum sativum*	CYP92A6/DDWF1	Kang et al., 2001
C3 oxidase	*Arabidopsis thaliana*	CYP90A1/CPD	Szekeres et al., 1996; Ohnishi et al., 2012
	*Oryza sativa*	CYP90A3/4(OsCPD1/2)	Sakamoto and Matsuoka, 2006
		CYP90D2/OsD2*	Hong et al., 2003; Li et al., 2013
	*Hordeum vulgare*	HvCPD	Dockter et al., 2014
C3 reductase	*Oryza sativa*	CYP724B1/OsD11*	Tanabe et al., 2005
C5 reductase	*Arabidopsis thaliana*	DET2	Chory et al., 1991; Fujioka et al., 1997; Noguchi et al., 1999
	*Glycine max*	GmDET2a/b	Huo et al., 2018
	*Gossypium hirsuturm*	GhDET2	Luo et al., 2007
	*Cucumis sativus*	CsDET2	Hou et al., 2017
	*Pisum sativum*	LK	Nomura et al., 2004
	*Pharbitis nil*	PnDET2	Suzuki et al., 2003
C6 oxidase	*Arabidopsis thaliana*	CYP85A1/2 (BR6ox1/2)	Shimada et al., 2001
	*Lycopersicon esculentum*	CYP85A1(DWARF)/A3	Bishop et al., 1999; Shimada et al., 2001
	*Pisum sativum*	PsCYP85A1/6	Jager et al., 2007
	*Oryza sativa*	OsDWARF/BRD1	Hong et al., 2002; Mori et al., 2002
	*Hordeum vulgare*	HvBRD	Dockter et al., 2014
	*Cucumis sativus*	SCP1/CsCYP85A1	Wang et al., 2017
	*Brachypodium distachyon*	BdBRD1	Xu et al., 2015
	*Zea mays*	ZmBRD1	Makarevitch et al., 2012
	*Populus trichocarpa*	PtCYP85A3	Jin et al., 2017

Enzymes marked by an asterisk are those with controversial functions.

#### Hydroxylation at C22, C23, and C2

There are at least five C22 hydroxylation reactions in the BR biosynthesis pathway, including campesterol to (22*S*)-22-hydroxy-campesterol, (24*R*)-ergost-4-en-3-one to (22*S*, 24*R*)-22-hydroxy-ergost-4-en-3-one, (24*R*)-5α-ergostan-3-one to (22*S*, 24*R*)-22-hydroxy-5α-ergostan-3-one, CN to 6-deoxocathasterone, and 6-oxocampestanol to cathasterone (Choe et al., 1998; Fujita et al., 2006; Ohnishi et al., 2006c). Although all these C22 hydroxylation reactions are catalyzed by the same cytochrome P450 monooxygenases in different plants, they prefer to take campesterol rather than others as a substrate (Choe et al., 1998; Fujita et al., 2006; Ohnishi et al., 2006c). In *Arabidopsis*, CYP90B1, a cytochrome P450 monooxygenase also known as DWARF4 (DWF4), is mainly responsible for these reactions (Choe et al., 1998; Fujita et al., 2006). CYP724A1 function at least partially as a C22 hydroxylase, since its overexpression can restore the deficiency caused by *dwf4* mutation (Zhang et al., 2012). Homologs of CYP90B1/DWF4 and CYP724A1 in different plant species, such as CYP90B2/OsDWF4 and CYP724B1/OsD11 in rice, CYP90B3 and CYP724B2 in tomato, CYP90B2/ZmDWF4 in maize, StDWF4 in potato (*Solanum tuberosum* L.), and PtoDWF4 in *Populus tomentosa*, were also found to possess similar biological functions (Ohnishi et al., 2006c; Sakamoto et al., 2006; Liu et al., 2007; Shen et al., 2018; Zhou et al., 2018). The C22 hydroxylation is considered as a rate-limiting step in the BR biosynthesis pathway possibly due to a low *DWF4* expression level that cannot effectively catalyze the reaction (Choe et al., 1998). This makes DWF4 an ideal target for manipulating BR biosynthesis to regulate growth and stress adaptation in modern agriculture (Choe et al., 2001; Kim et al., 2006; Sakamoto et al., 2006; Sahni et al., 2016; Li et al., 2018; Zhou et al., 2018).

Six C23 hydroxylation reactions, including (22*S*)-22-hydroxy-campesterol to (22*R*, 23*R*)-22, 23-dihydroxy-campesterol, (22*S*, 24*R*)-22-hydroxy-ergost-4-en-3-one to (22*R*, 23*R*)-22, 23-dihydroxy-campest-4-en-3-one, (22*S*, 24*R*)-22-hydroxy-5α-ergostan-3-one to 3-dehydro-6-deoxoteasterone, 3-*epi*-6-deoxocathasterone to 6-deoxotyphasterol, 6-deoxocathasterone to 6-deoxoteasterone, and cathasterone to teasterone, were identified in the BR biosynthesis pathway (Ohnishi et al., 2006b; Sakamoto et al., 2012). (22*S*, 24*R*)-22-hydroxy-5α-ergostan-3-one and 3-*epi*-6-deoxocathasterone are two favorable substrates for the C23 hydroxylases in plants, leading to a shortcut with two steps less than other biosynthetic routes (Ohnishi et al., 2006b; Sakamoto et al., 2012). The C23 hydroxylases are also members of cytochrome P450 monooxygenases, such as CYP90C1/ROTUNDIFOLIA3 (ROT3) and CYP90D1 in *Arabidopsis*, CYP90D2/OsD2 and CYP90D3 in rice, and CYP90C2/DUMPY (DPY) in tomato (Koka et al., 2000; Ohnishi et al., 2006b; Sakamoto et al., 2012).

The C2 hydroxylation steps, converting 6-deoxotyphasterol to 6-deoxocastasterone and typhasterol to castasterone located in the late and the early C6 oxidation pathways respectively, have only been elucidated in pea (Kang et al., 2001). A dark-induced cytochrome P450, named as DARK-INDUCED DWF-LIKE PROTEIN 1 (DDWF1), is activated by a small G protein PRA2 and then to catalyze the C2 hydroxylation reactions in the BR biosynthetic pathway (Kang et al., 2001).

#### Oxidation and Reduction at C3

At least twice redox reactions at C3 position were found in the BR biosynthetic pathway. The big difference between the dominant CN-independent route from others is that it contains one less C3 redox reaction (Ohnishi et al., 2012). The first time of C3 oxidation reactions include campesterol to (24*R*)-ergost-4-en-3-one, (22*S*)-22-hydroxy-campesterol to (22*S*, 24*R*)-22-hydroxy-ergost-4-en-3-one, and (22*R*, 23*R*)-22, 23-dihydroxycampesterol to (22*R*, 23*R*)-22, 23-dihydroxy-campest-4-en-3-one. Conversions from 6-deoxoteasterone to 3-dehydro-6-deoxoteasterone and from teasterone to 3-dehydroteasterone in the late and the early C6 oxidation pathways, respectively, are the second C3 oxidation reactions. In *Arabidopsis*, CYP90A1/CPD is responsible for the C3 oxidation and has a broad substrate specificity. Three of the five intermediates, (22*S*)-22-hydroxy-campesterol, (22*R*, 23*R*)-22, 23-dihydroxycampesterol, and 6-deoxoteasterone, can be converted to their respective 3-dehydro derivatives by CYP90A1/CPD, whereas, its preferred substrate is (22*S*)-22-hydroxy-campesterol (Szekeres et al., 1996; Ohnishi et al., 2012). Rice CYP90A3/OsCPD1 and CYP90A4/OsCPD2 were predicted to perform the similar function as *Arabidopsis* CYP90A1/CPD based on their high sequence similarity (Sakamoto and Matsuoka, 2006). However, rice CYP90D2/OsD2 is considered as the C3 oxidase for 6-deoxoteasterone and teasterone by two research groups, while another research group demonstrated that it functions redundantly with CYP90D3 as a C23 hydrolase (Hong et al., 2003; Sakamoto et al., 2012; Li et al., 2013).

C3 reductions include conversions from (24*R*)-5α-ergostan-3-one to campestanol, (22*S*, 24*R*)-22-hydroxy-5α-ergostan-3-one to 6-deoxocathasterone or 3-*epi*-6-deoxocathasterone, 3-dehydro-6-deoxoteasterone to 6-deoxotyphasterol, and 3-dehydroteasterone to typhasterol. In rice, CYP724B1/OsD11 is originally reported as the C3 reductase to produce 6-deoxotyphasterol and typhasterol (Tanabe et al., 2005). However, a different research group declared that it catalyzes the C22 hydroxylation together with CYP90B2/OsDWF4 (Sakamoto et al., 2006). The BR C3 reductase in *Arabidopsis* model plant is yet to be identified in the future.

#### C5 Reduction

C5 reduction is an early reaction step in the BR biosynthesis pathway, leading to the formation of (24*R*)-5α-ergostan-3-one, (22*S*, 24*R*)-22-hydroxy-5α-ergostan-3-one, and 3-dehydro-6-deoxoteasterone from (24*R*)-ergost-4-en-3-one, (22*S*, 24*R*)-22-hydroxy-ergost-4-en-3-one, and (22*R*, 23*R*)-22, 23-dihydroxy-campest-4-en-3-one, respectively. A steroid 5α-reductase, named as DEETIOLATION 2 (DET2), is responsible for the C5 reduction in *Arabidopsis* (Chory et al., 1991; Li et al., 1996; Fujioka et al., 1997; Li et al., 1997; Noguchi et al., 1999). Paralogs of DET2 in different plant species have also been identified, such as in soybean, cotton, cucumber, pea, and morning glory (Suzuki et al., 2003; Nomura et al., 2004; Luo et al., 2007; Hou et al., 2017; Huo et al., 2018).

#### C6 Oxidation and Baeyer-Villiger Oxidation

C6 oxidation converts the 6-deoxo BR intermediates in the late C6 oxidation pathway to corresponding 6-oxo compounds in the early C6 oxidation pathway. Although several pairs of substates and products seem to occur naturally in a number of plant species, only the conversion from 6-deoxotyphasterol to typhasterol and from 6-deoxocastasterone to castasterone have been verified in *Arabidopsis* and rice (Shimada et al., 2001; Mori et al., 2002). Conversions from 6-deoxoteasterone to teasterone and from 3-dehydro-6-deoxoteasterone to 3-dehydroteasterone were also thought to occurr but remain tentative in *Arabidopsis* and possibly other plants (Shimada et al., 2001). Whereas, in tomato, conversion from 6-deoxocastasterone to castasterone seems to be the only major C6 oxidation pathway (Bishop et al., 1999; Shimada et al., 2001). The C6 oxidases encoded by cytochrome P450s have been identified in different plant species, such as CYP85A1/2 (also name as BR6ox1/2) in *Arabidopsis*, DWARF/CYP85A1 and CYP85A3 in tomato, PsCYP85A1 and PsCYP85A6/LKE in pea, OsDWARF/BRD1 in rice, SCP1/CsCYP85A1 in cucumber, HvBRD in barley, BdBRD1 in *Brachypodium distachyon*, ZmBRD1 in maize, PtCYP85A3 in *Populus trichocarpa*, and so on (Bishop et al., 1999; Shimada et al., 2001; Hong et al., 2002; Mori et al., 2002; Jager et al., 2007; Makarevitch et al., 2012; Dockter et al., 2014; Xu et al., 2015; Jin et al., 2017; Wang et al., 2017). It should be noted that the C6 oxidation is also a rate-limiting step in the BR biosynthesis pathway (Nomura et al., 2001).

The Baeyer-Villiger oxidation creates a lactone at ring B of the steroid back­bone, leading to the formation of BL from CS in *Arabidopsis* and tomato but not in rice (Kim et al., 2005b; Nomura et al., 2005; Kim et al., 2008). Consistently, there is only one copy of CYP85A gene in rice, while there are two copies of CYP85As in *Arabidopsis* and tomato genomes. It has been elucidated that the extra CYP85A enzymes, CYP85A2/BR6ox2 in *Arabidopsis* and CYP85A3 in tomato, are responsible for the Baeyer-Villiger oxidation (Kim et al., 2005b; Nomura et al., 2005).

### Regulation of BR Biosynthesis

**Figure 3:**
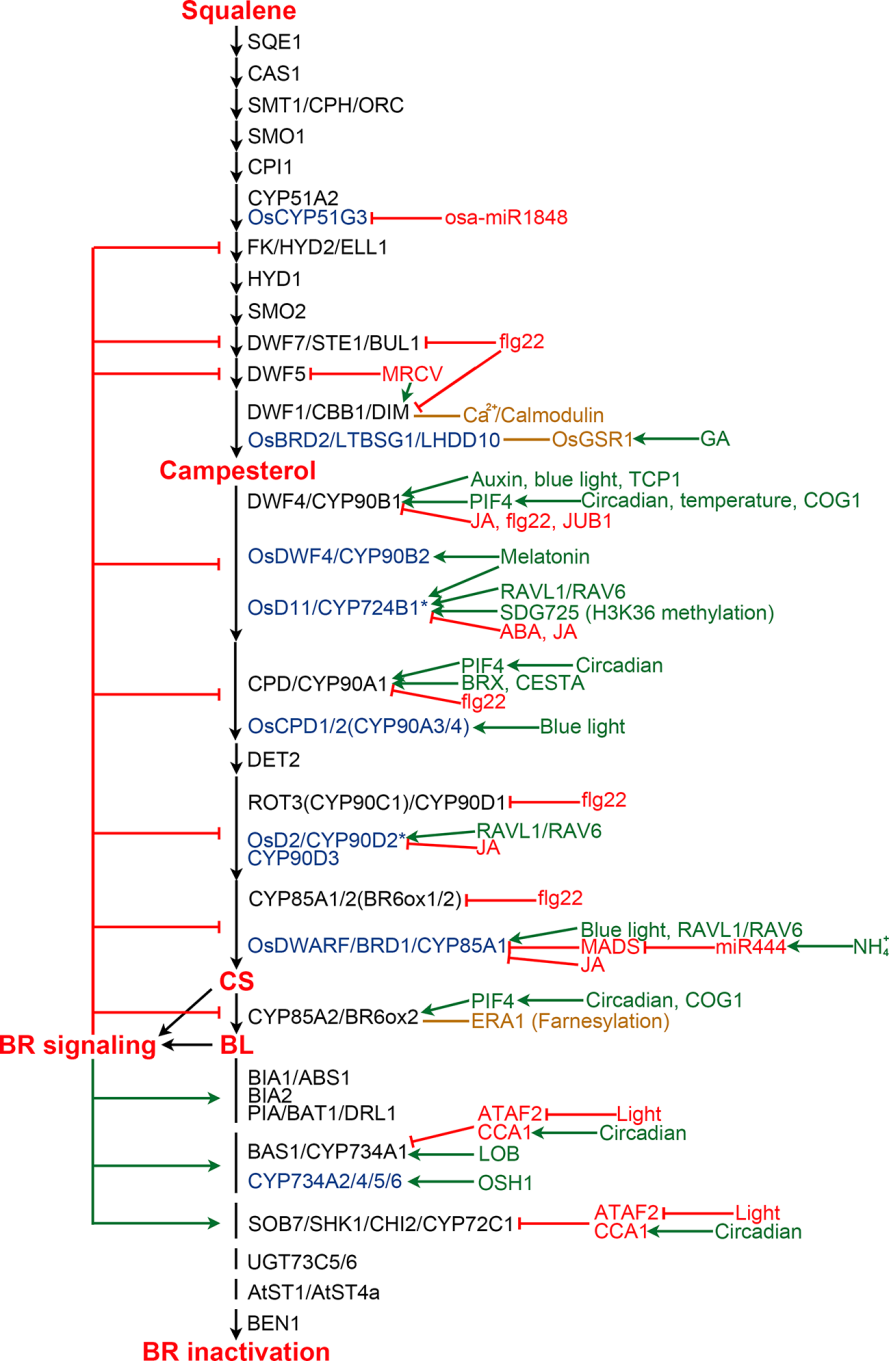
Regulation of BR biosynthesis and catabolism in Arabidopsis and rice. BR biosynthesis pathway is shown from squalene to BL. Enzymes from Arabidopsis and rice are shown in black and blue colors, respectively. Enzymes marked by an asterisk are those with controversial functions. Enzymes from other plants are list in **Table 2** and **Table 3**. Green and red arrows indicate positive and negative regulation, respectively. Orange lines represent protein-protein interaction.

BR biosynthesis is inhibited by the end product, CS or BL, *via* a feedback loop (**Figure 3**). Exogenous application of BL leads to down-regulation of multiple BR biosynthetic genes, while BR biosynthesis inhibitors induce the expression of these genes, suggesting feedback transcriptional regulation occurs at multiple steps of the BR biosynthesis pathway (Mathur et al., 1998; Hong et al., 2002; Hong et al., 2003; Tanabe et al., 2005; Tanaka et al., 2005). Now, it is clear that perception of BL by its receptor BRI1 and coreceptor BAK1 ultimately leads to the activation of a group of transcription factors, including BES1 and BZR1, in the nucleus (Li and Chory, 1997; Li et al., 2002; Wang et al., 2002; Yin et al., 2002). BES1 and BZR1 not only regulate the expression of thousands of genes involved in diverse processes during plant growth and development, but also are responsible for the feedback inhibition *via* directly binding to the promoter regions of multiple BR biosynthesis genes to repress their expression (He et al., 2005; Sun et al., 2010; Yu et al., 2011).

The BR biosynthesis pathway is regulated by various internal signaling molecules and by its end products (**Figure 3**). For example, auxin induces *DWF4* expression to increase endogenous BR level in *Arabidopsis* roots, partially by repressing the binding of BZR1 to the *DWF4* promoter (Chung et al., 2011). BREVIS RADIX (BRX) mediates auxin action on BR biosynthesis through activating the *CPD* expression in *Arabidopsis* (Mouchel et al., 2006). Gibberellins (GAs) have also been reported to play roles in regulating BR biosynthesis. OsGSR1, a GAST member (a GA-stimulated transcript) induced by GA and repressed by BR at a transcription level, interacts with DIM/DWF1 to modulate the BR level in rice (Wang et al., 2009). SPINDLY, an *O*-linked *N*-acetylglucosamine transferase negatively regulating GA signaling in rice, represses BR biosynthesis *via* an unknown mechanism (Shimada et al., 2006). JUNGBRUNNEN1 (JUB1), a NAC transcriptional regulator, acts at the nexus of BR-GA network by regulating a complex transcriptional module composed of key components of GA and BR pathways including *GA3ox1*, *DWF4* and the DELLA genes *GAI* and *RGL1* (Shahnejat-Bushehri et al., 2016). Moreover, the functional mechanism of JUB1 in regulating BR/GA biosynthesis and signaling is considerably conserved across species (Shahnejat-Bushehri et al., 2017). Jasmonic acid and abscisic acid were found to inhibit the expression of BR biosynthetic genes to antagonize BR functions (Ren et al., 2009; Gan et al., 2015; Li et al., 2019). Melatonin also plays a role in regulating BR biosynthesis. Block of its biosynthesis results in a decreased BR level in rice, while exogenously applied melatonin induces the expression of BR biosynthesis genes (Hwang and Back, 2018; Hwang and Back, 2019; Lee and Back, 2019).

Pieces of evidence support that light plays an important role in regulating BR biosynthesis. In rice aerial tissues, blue light promotes expression of *CYP85A1*/*BRD1*/*OsDWARF* and *OsCYP90A3*/*4*, thereby increasing CS level. While, far-red light, instead of blue light or red light, positively regulate BR biosynthesis in rice roots (Asahina et al., 2014). However, in *Arabidopsis*, blue light perception in aerial tissues enhances *DWF4* accumulation in the root tips (Sakaguchi and Watanabe, 2017; Sakaguchi et al., 2019). Moreover, the expression levels of *Arabidopsis CPD* and *CYP85A2*/*BR6ox2* display complex diurnal patterns (Bancos et al., 2006). A recent study revealed that BES1 inhibits the expression of BR biosynthesis genes during the day, while elevated PIF4 competes for BES1 resulting in de-repressed BR biosynthesis at dawn (Martinez et al., 2018). In addition, it was found that PIF5 acts redundantly with PIF4 to positively regulate BR biosynthesis. COG1, a Dof type transcription factor negatively regulating phytochrome signaling, can directly promote the expression of *PIF4* and *PIF5*. PIF4 and PIF5 then directly bind to the promoters of *DWF4* and *CYP85A2*/*BR6ox2* to enhance their expression, resulting in elevated levels of endogenous BRs (Park et al., 2003; Wei et al., 2017). It was demonstrated that PIF4 also activates the expression of BR biosynthesis genes in response to elevated temperatures to promote thermomorphogenic hypocotyl growth (Maharjan and Choe, 2011; Martinez et al., 2018).

BR biosynthesis is highly regulated by different environmental stimuli as well as light. For instance, ammonium (NH_4_^+^), one of the major nitrogen resources for plants, induces the accumulation of miR444, which then positively regulates rice BR biosynthesis *via* inhibiting its MADS-box targets and subsequently activating *OsBRD*1 expression (Jiao et al., 2020). Calmodulin, a Ca^2+^ sensor protein which plays an essential role in sensing and transducing environmental stimuli, can interact with DWF1 in a Ca^2+^-dependent manner and control its function to regulate BR biosynthesis (Du and Poovaiah, 2005). Bacterial flagellin 22 triggers plant immunity responses, resulting in reduced expression of several BR biosynthetic genes, including *CPD*, *DWF4*, *BR6ox1/2*, *ROT3*, *DWF1*, and *DWF7* in *Arabidopsis* (Jimenez-Gongora et al., 2015). *Mal de Río Cuarto virus* (MRCV) causes severe diseases in several monocotyledonous crops. It was found that MRCV infection causes the up-regulation of *DIM*/*DWF1* but the down-regulation of *DWF5*, and significantly increased amount of BL in wheat (de Haro et al., 2019).

Several additional components regulating BR biosynthesis were identified from different plant species. However, their upstream signaling is yet to be elucidated in the future. In *Arabidopsis*, TCP1, a basic helix loop helix (bHLH) transcription factor, can directly bind to the promoter of *DWF4* to enhance its expression and promotes BR biosynthesis (Guo et al., 2010; An et al., 2011; Gao et al., 2015). CESTA, another bHLH transcription factor, positively regulates BR biosynthesis *via* promoting the expression of *CPD* (Poppenberger et al., 2011). Farnesylation, a post-translational modification, of *Arabidopsis* CYP85A2/BR6ox2 was found to be essential for its subcellular localization and function. Loss of CYP85A2/BR6ox2 farnesylation results in reduced BL accumulation, similar to the mutation of CYP85A2/BR6ox2 (Northey et al., 2016; Jamshed et al., 2017). In rice, RAVL1 and RAV6, two homologous B3 transcription factors, mediate activation of both OsBRI1 and the BR biosynthetic genes that have antagonistic actions on BR levels to ensure the basal activity of the BR signaling and biosynthetic pathways (Il Je et al., 2010; Zhang et al., 2015). Rice microRNA osa-miR1848 mediates *OsCYP51G3* mRNA cleavage to regulate phytosterol and BR biosynthesis (Xia et al., 2015). SDG725, a H3K36 methyltransferase from rice, modulates the expression of *OsD11*, suggesting an important role of H3K36 methylation on BR biosynthesis (Sui et al., 2012). In addition, both SLG and XIAO, predicted to be a BAHD acyltransferase-like protein and a leucine-rich repeat protein like kinase (LRR-RLK), respectively, function as the positive regulators of BR biosynthesis *via* unknown mechanisms (Jiang et al., 2012; Feng et al., 2016). In wheat, TaSPL8 binds to the promoter of *CYP90D2/OsD2* to activate its expression and regulate leaf angle (Liu et al., 2019). In cotton, GhFP1, a bHLH transcription factor, directly binds to the promoters of *GhDWF4* and *GhCPD* to activate their expression (Liu et al., 2020b). In apple, MdWRKY9 directly represses *MdDWF4* transcription to inhibit BR biosynthesis (Zheng et al., 2018). MdNAC1 negatively modulates BR production probably by inhibiting the expression of *MdDWF4* and *MdCPD* (Jia et al., 2018).

## Catabolism

### BR Catabolism

Endogenous bioactive levels of BRs are also controlled by their catabolic processes. BR catabolism leads to decreased levels of bioactive BRs and attenuated signaling output. Elucidation of BR catabolism can help us, from a different aspect, to understand how plants regulate BR homeostasis for their optimal growth, development and environmental adaptations. Diverse modifications of BRs were revealed by various feeding experiments and analytic chemistry analyses, such as epimerization of C2 and C3 hydroxy groups, hydroxylation of C20, C25, and C26, side chain cleavage; sulfonation of C22; conjugation with fatty acids or glucose; acylation; demethylation; and so on (Fujioka and Yokota, 2003). At present, several BR inactivating reactions and some of their corresponding enzymes have been demonstrated in plants (**Table 3**). In *Arabidopsis*, at least 10 BR inactivating enzymes with different or similar biochemical mechanisms have been identified. Overexpression of these BR catabolic genes leads to BR deficiency, whereas loss of function of these enzymes results in elevated amounts of BL or CS in plants.

**Table 3:** BR metabolism enzymes in different plant species.

Function	Species	Name	Reference
Acyltransferase	*Arabidopsis thaliana*	BIA1/ABS1	Roh et al., 2012; Wang et al., 2012
		BIA2	Zhang and Xu, 2018
		PIZ/BAT1/DRL1	Schneider et al., 2012; Choi et al., 2013; Zhu et al., 2013
C26 hydroxylase	*Arabidopsis thaliana*	BAS1/CYP734A1	Neff et al., 1999; Turk et al., 2003
	*Lycopersicon esculentum*	CYP734A7	Ohnishi et al., 2006a
	*Oryza sativa*	CYP734A2/4/5/6	Sakamoto et al., 2011
	*Gossypium hirsuturm*	PAG1	Yang et al., 2014
	*Daucus carota*	DcBAS1	Que et al., 2019
Sulfotransferase	*Brassica napus*	BNST3/4	Rouleau et al., 1999; Marsolais et al., 2004
	*Arabidopsis thaliana*	AtST1/AtST4a	Marsolais et al., 2007
Glycosyltransferase	*Arabidopsis thaliana*	UGT73C5	Poppenberger et al., 2005
		UGT73C6	Husar et al., 2011
Unknown	*Arabidopsis thaliana*	CYP72C1/SOB7/SHK1/CHI2	Nakamura et al., 2005; Takahashi et al., 2005; Turk et al., 2005
Unknown	*Arabidopsis thaliana*	BEN1	Yuan et al., 2007

#### Hydroxylases

C26 hydroxylation is a relatively well characterized way of BR inactivation. *Arabidopsis* BAS1/CYP734A1 (formerly named CYP72B1) is the first reported BR C26 hydroxylase as revealed by the feeding experiment. It is able to convert both CS and BL to their C26 hydroxylated derivatives (Neff et al., 1999; Turk et al., 2003). Such modification possibly prevents the side chain of BRs from fitting into the binding pocket of the receptor, BRI1 (Hothorn et al., 2011; She et al., 2011). Tomato CYP734A7 can also convert CS and BL to their hydroxylated products, respectively (Ohnishi et al., 2006a). CYP734A orthologs from rice control endogenous bioactive levels of BRs by metabolizing both CS and its precursors (Sakamoto et al., 2011; Thornton et al., 2011). It is noteworthy that rice CYP734As can catalyze not only the hydroxylation but also the second and the third oxidations to produce aldehyde and carboxylate groups at C26 (Sakamoto et al., 2011). PAG1 from cotton and DcBAS1 from carrot may also inactivate bioactive BRs in a way similar to that of the *Arabidopsis* BAS1 (Yang et al., 2014; Que et al., 2019).

CYP72C1/SOB7/SHK1/CHI2, a homolog of BAS1/CYP734A1, was identified by three independent research groups in the same year. It acts redundantly with BAS1/CYP734A1 to modulate *Arabidopsis* photomorphogenesis and BR inactivation processes (Nakamura et al., 2005; Takahashi et al., 2005; Turk et al., 2005). However, CYP72C1 prefer to act on BR immediate precursors *via* an uncharacterized mechanism, which is different from CYP734A members that can inactivate both BL and CS through C26 hydroxylation (Thornton et al., 2010).

#### Glycosyltransferases

Glucosylation is one of the important regulatory mechanisms controlling hormone homeostasis *in planta*. CS and BL can be glucosylated at different positions. C2-, C3-, C22-, and C23-glucosylation of CS, and C2-, C3-, and C23-glucosylation of BL were confirmed, although the glucosylation profiles varied in different plant species (Soeno et al., 2006). 23-*O*-glucosylation of CS or BL was found to be predominant in *Arabidopsis*, which is catalyzed by two homologous UDP-glycosyltransferases named UGT73C5 and UGT73C6 (Poppenberger et al., 2005; Husar et al., 2011). Overexpression of *UGT73C5* or *UGT73C6* leads to a BR-deficient phenotype in *Arabidopsis*. Since these two functionally redundant genes are tightly linked, it is impossible to get high-order null mutant with traditional genetics to support the biochemical analysis results at the time when it was first published. Of course, using a CRISPR-Cas9 approach can solve such a problem at present time. In addition, enzymes mediating C2-, C3-, and C22- glucosylation of BRs in plants are still unknown.

#### BEN1, a Putative Reductase

BRI1-5 ENHANCED1 (BEN1) is also involved in BR inactivation in *Arabidopsis* (Yuan et al., 2007). Although the detailed biochemical mechanism has not been elucidated, strong genetic evidence supports that BEN1 functions as a BR inactivating enzyme. Gain of function of BEN1 severely enhances the *bri1-5* defective phenotype, while loss of function of BEN1 leads to an organ-elongation phenotype. Since *BEN1* encodes a dihydroflavonol 4-reductase (DFR)-like protein, it is hypothesized that BEN1 functions as a reductase to convert 6-oxo BR intermediates to their 6-deoxo counterparts (Yuan et al., 2007). It is noteworthy that the intronic T-DNA insertion in the *ben1-1* mutant is epigenetically regulated (Sandhu et al., 2013).

#### Acyltransferases

Three acyltransferases were found to decrease endogenous bioactive levels of BRs likely *via* different biochemical mechanisms in *Arabidopsis*. BRASSINOSTEROID INACTIVATOR 1 (BIA1)/ABNORMAL SHOOT1 (ABS1), a BAHD acyltransferase in *Arabidopsis*, was isolated by two independent research groups. Activation tagged mutants or transgenic plants overexpressing *BIA1*/*ABS1* display reduced levels of endogenous BRs and BR-deficient phenotypes that can be rescued by exogenous application of active BRs, indicating a possible role of BIA1/ABS1 in maintaining BR homeostasis (Roh et al., 2012; Wang et al., 2012). A more recent study demonstrated that BIA1 uses acetyl-CoA as a donor substrate to acylate CS, leading to the formation of monoacetylated and diacetylated CS (Gan et al., 2020). BIA2, a homolog of BIA1/ABS1 in *Arabidopsis*, also plays a role in BR inactivation possibly *via* the esterification of certain BRs (Zhang and Xu, 2018). PIZZA (PIZ)/BR-RELATED ACYLTRANSFERASE1 (BAT1)/DWARF AND ROUND LEAF1 (DRL1), an acyltransferase in *Arabidopsis*, was found to regulate BR homeostasis probably by converting BR intermediates into acylated inactive conjugates (Schneider et al., 2012; Choi et al., 2013; Zhu et al., 2013).

#### Sulfotransferases

BNST3 and BNST4, two homologous steroid sulfotransferases from *Brassica napus*, catalyze the *in vitro O*-sulfonation of BRs as well as mammalian estrogenic steroids and hydroxysteroids (Rouleau et al., 1999; Marsolais et al., 2004). They are stereospecific for 24-epiBRs, with a preference for 24-epicathasterone, an intermediate in the biosynthesis of 24-epiBL, which is different from other known metabolic enzymes that utilize CS and BL as substrates. However, BNST3 and BNST4 were also thought to be involved in BR inactivation since sulfonation of 24-epiBL leads to the absence of its biological activity in the bean second internode bioassay (Rouleau et al., 1999; Marsolais et al., 2004). AtST1, an *Arabidopsis* ortholog of BNST3 and BNST4, displays a similar specificity toward 24-epiBRs. Whereas, AtST4a, another steroid sulfotransferase from *Arabidopsis*, is specific for bioactive BR compounds (Marsolais et al., 2007). Genetic evidence to support the significance of these sulfotransferases in BR inactivation is still lacking (Sandhu and Neff, 2013).

### Regulation of BR Catabolism

Plants evolved various mechanisms to control BR catabolism (**Figure 3**). Feedback regulation of key BR catabolic genes is one of these mechanisms. It was found that BL induces the expression of several BR catabolic genes, including *BIA1/ABS1*, *BIA2*, *PIZ/BAT1/DRL1*, *BAS1/CYP734A1*, and *SOB7/SHK1/CHI2* in *Arabidopsis*, *PAG1* in cotton, and *DcBAS1* in carrot (Tanaka et al., 2005; Roh et al., 2012; Zhu et al., 2013; Yang et al., 2014; Zhang and Xu, 2018; Que et al., 2019).

Besides the end products of the BR biosynthetic pathway, other phytohormones were also found to regulate the expression of the BR catabolic genes. For example, the expression of *BNST3/4* can be induced by salicylic acid (Rouleau et al., 1999). The expression of *PIZ/BAT1/DRL1* is induced by auxin but repressed by abscisic acid (Choi et al., 2013; Zhu et al., 2013). Moreover, ARF7, an auxin responsive transcription factor, can directly inhibit the expression of *BAS1/CYP734A1* to increase endogenous BR contents in *Arabidopsis*, providing sufficient evidence that auxin regulates BR catabolism (Youn et al., 2016).

Most of the abovementioned BR catabolic genes show different expression patterns under light and in darkness, indicating an important role of light in maintaining BR homeostasis by regulating the catabolic reactions. However, little is known about the detailed mechanisms. It has been found that PHYB, a red/far red-absorbing phytochrome, modulates *BAS1* expression in *Arabidopsis* shoot apex to inhibit phase transition (Sandhu et al., 2012). ATAF2, an *Arabidopsis* NAC transcription factor suppressed by light at a transcription level, modulates BR inactivation *via* directly binding to the promoter of *BAS1/CYP734A1* and *SOB7/SHK1/CHI2* to repress their expression (Peng et al., 2015). A more recent study demonstrated that CIRCADIAN CLOCK ASSOCIATED 1 (CCA1), a MYB transcription factor, interacts with ATAF2 and directly regulates the oscillation expression of *BAS1/CYP734A1* and *SOB7/SHK1/CHI2* (Peng and Neff, 2020).

Two more transcription factors were found to be involved in regulating BR catabolism. However, the upstream signaling is unknown. LATERAL ORGAN BOUNDARIES (LOB) activates *BAS1/CYP734A1* expression *via* directly binding to its promoter and consequently decrease BR accumulation to limit growth in *Arabidopsis* organ boundaries (Bell et al., 2012). OSH1, a KNOX transcription factor, promotes the expression of three homologous BR catabolic genes, *CYP734A2*, *CYP734A4*, and *CYP734A6*, to control local bioactive BR levels in rice shoot apical meristems (Tsuda et al., 2014).

## General Conclusion

It has been about fifty years since BL was first discovered from *Brassica napus* pollen grains (Mitchell et al., 1970). Significant progress has been made in our understanding of BR biosynthesis and catabolism. Although the BR biosynthesis pathway displays a metabolic grid, the most dominant and efficient shortcut was established, with only eight and seven steps in *Arabidopsis* and rice, respectively (**Figure 2**). Moreover, enzymes catalyzing each reaction in the BR biosynthetic pathway, except for the C2 hydroxylation and the C3 redox reaction, have been identified by using analytical chemistry and molecular genetic approaches. Structural and physiological studies revealed that C2 and C3 positions are important for BR activity and perception by its receptor and coreceptor (Hothorn et al., 2011; She et al., 2011; Sun et al., 2013). Therefore, identification of C2 hydroxylase and C3 oxidase/reductase is essential for clarifying the whole picture of BR biosynthesis. CYP92A6/DDWF1 from pea was identified as the C2 hydroxylase, providing reference for study of C2 hydroxylase in *Arabidopsis*, rice, and other higher plants (Kang et al., 2001). As revealed by feeding experiments or anticipated from naturally occurring metabolites, various BR metabolic reactions were found (Fujioka and Yokota, 2003). However, little is known about the corresponding enzymes and the underlying mechanisms. Moreover, knowledge about how BR biosynthesis and catabolism are regulated, especially in a specific organ or tissue, by diverse internal and external cues is still very limited. Elucidating the mechanisms regulating BR homeostasis can help us to generate high-yield transgenic crops *via* manipulating bioactive BR contents. For example, modulating the expression of C22 hydroxylase, catalyzing the rate-limiting step in BR biosynthesis pathway, in different plant species indeed resulted in increased vegetative growth, yield, and tolerance (Choe et al., 2001; Sakamoto et al., 2006; Guo et al., 2010; Sakaguchi and Watanabe, 2017; Li et al., 2018; Zhou et al., 2018). It might not be possible to obtain optimal BR effects for all of the agronomic traits, since BRs control many aspects of plant growth and development, and responses to biotic and abiotic stresses. However, even if a subset of these traits can be improved by BRs, the accomplishment will be significant.

## Author Contributions

ZW prepared the manuscript. JL revised the manuscript.

## Funding

We are grateful for the support from National Natural Science Foundation of China (grant no. 31720103902, 31530005, 31700245), the 111 Project (grant no. B16022), and Fundamental Research Funds for the Central Universities (grant no. lzujbky-2020-32 from Lanzhou University).

## Conflict of Interest

The authors declare that the research was conducted in the absence of any commercial or financial relationships that could be construed as a potential conflict of interest.
